# DNA Repair Protein XRCC1 Stimulates Activity of DNA Polymerase λ under Conditions of Microphase Separation

**DOI:** 10.3390/ijms25136927

**Published:** 2024-06-25

**Authors:** Natalia A. Lebedeva, Rashid O. Anarbaev, Ekaterina A. Maltseva, Maria V. Sukhanova, Nadejda I. Rechkunova, Olga I. Lavrik

**Affiliations:** Institute of Chemical Biology and Fundamental Medicine (ICBFM), Siberian Branch of the Russian Academy of Sciences (SB RAS), Novosibirsk 630090, Russia; nataleb@niboch.nsc.ru (N.A.L.); anarbaev@niboch.nsc.ru (R.O.A.); 060179@mail.ru (E.A.M.); mary@niboch.nsc.ru (M.V.S.); nadyarec@niboch.nsc.ru (N.I.R.)

**Keywords:** XRCC1, DNA polymerase λ, microphase separation, base excision repair

## Abstract

Non-membrane compartments or biomolecular condensates play an important role in the regulation of cellular processes including DNA repair. Here, an ability of XRCC1, a scaffold protein involved in DNA base excision repair (BER) and single-strand break repair, to form protein-rich microphases in the presence of DNA duplexes was discovered. We also showed that the gap-filling activity of BER-related DNA polymerase λ (Pol λ) is significantly increased by the presence of XRCC1. The stimulation of the Pol λ activity was observed only at micromolar XRCC1 concentrations, which were well above the nanomolar dissociation constant determined for the XRCC1–Pol λ complex and pointed to the presence of an auxiliary stimulatory factor in addition to protein–protein interactions. Indeed, according to dynamic light scattering measurements, the stimulation of the Pol λ activity by XRCC1 was coupled with microphase separation in a protein–DNA mixture. Fluorescence microscopy revealed colocalization of Pol λ, XRCC1, and gapped DNA within the microphases. Thus, stimulation of Pol λ activity is caused both by its interaction with XRCC1 and by specific conditions of microphase separation; this phenomenon is shown for the first time.

## 1. Introduction

Base excision repair (BER) is the main DNA repair pathway protecting mammalian cells against single-base DNA damage and spontaneously arising apurinic/apyrimidinic (AP) sites [1]. BER is performed either via the short-patch (insertion of one nucleotide) or via the long-patch (insertion of several nucleotides) pathway. Initially, DNA polymerase β (Pol β) was characterized as a major polymerase involved in BER [2,3,4,5,6]. Pol β belongs to the X-family and has two enzymatic activities, namely DNA polymerase and 5′-deoxyribose-5-phosphate (5′-dRP) lyase. Nevertheless, growing evidence indicates that in BER, there are alternate polymerases that can substitute for Pol β in the repair process [7]. DNA polymerase λ (Pol λ; an X-family DNA polymerase) is the closest homolog of Pol β and can function as a backup polymerase in BER [7,8,9,10,11,12]. Pol β and Pol λ have high amino acid sequence homology with conservation of all critical residues involved in DNA and dNTP binding [7,13,14,15,16]. Just like Pol β, Pol λ possesses DNA polymerase and 5′-dRP lyase activities and effectively performs gap-filing DNA synthesis; this protein includes a Pol β-like domain (an 8 kDa dRP lyase subdomain and a 31 kDa DNA polymerase subdomain) but also contains additional proline-serine-rich (SPR) and N-terminal BRCT domains, which are implicated in protein–protein interactions [7,8,9]. It was previously shown that these domains suppress the polymerase activity of the Pol λ [17,18], and the SPR domain confers the increase in the fidelity of Pol λ [19]. The presence of the BRCT domain appears to allow Pol λ to participate in the repair of double-stranded DNA breaks by non-homologous end-joining (NHEJ) via protein–protein interactions [8,9]. Thus, the Pol λ BRCT domain was found to mediate interactions with other protein factors necessary for the recognition and binding of broken DNA ends [8,9]. Moreover, the BRCT domain of Pol λ has sequence and structural differences compared to two previously studied homologs of family X polymerases, such as DNA polymerase μ (Pol μ) and Terminal deoxynucleotidyl transferase [20]. Thus, the dRP lyase activity of Pol λ and the feature in its structure in comparison with Pol μ allows it to function in both NHEJ and BER [21]. Summarizing all of the above, Pol λ can take part in different processes: BER or the repair of double-stranded breaks through non-homologous end-joining pathways [8,22,23,24,25].

Similar to Pol β [6,26], the functions of Pol λ in BER may be modulated by repair factors such as PARP1, PARP2 (members of the ADP-ribosyltransferases diphtheria toxin-like family [ARTDs]), and X-ray repair cross-complementing protein 1 (XRCC1). Some studies have shown that BER or single-strand break (SSB) repair factors such as PARP1, PARP2, and XRCC1 can modulate the DNA synthesis activity of Pol β in vitro [6,27,28,29]. The regulation of the Pol β-mediated DNA synthesis by PARP1 and/or PARP2 or XRCC1 has been reported to be a result of one of the following: (i) competition between Pol β and PARP1 and/or PARP2 for the binding to DNA repair intermediates containing breaks; (ii) the formation of an XRCC1–Pol β–DNA ternary complex [6,27,29]. On the other hand, the synthesis of poly(ADP-ribose) (PAR) catalyzed by PARP1 or PARP2 coupled with protein PARylation stimulates this process [6,29]. In contrast to PARP1 and PARP2, XRCC1 does not possess any enzymatic activity but interacts with all the enzymes participating in BER, e.g., Pol β, apurinic/apyrimidinic endonuclease 1 (APE1), polynucleotide kinase 3′-phosphatase (PNKP), DNA ligase III (Lig III), PARP1, and PARP2, thereby serving as a scaffold or “platform” for the assembly of repair complexes [30,31,32]. XRCC1 consists of an N-terminal domain, two BRCT domains (BRCTa and BRCTb), and two interdomain linker regions [30,33,34,35]. The XRCC1–enzyme interactions can result in a regulation of the activity of the respective enzymes, as shown to be the case for Pol β [26]. The presence of two BRCT domains (so-called protein–protein interaction modules [32]) creates preconditions for the interaction of XRCC1 with BRCT-containing Pol λ. Although Pol λ-dependent DNA repair synthesis has been intensively researched in numerous studies [10,11,12,36,37,38,39], the functional interaction between Pol λ and other BER factors is still not fully elucidated. 

In the present work, we examine specific features of functional interaction of Pol λ—or its truncated form lacking N-terminal BRCT and SPR domains (Pol Δλ)—with XRCC1 protein factor involved in BER regulation in comparison with Pol β. We found that full-length Pol λ interacts with XRCC1 with strong affinity, but the affinity of XRCC1 for Pol Δλ proved to be 15-fold weaker, suggesting that the Pol λ N-terminal BRCT and SPR domains play a pivotal part in the interaction with XRCC1. XRCC1 stimulated the activity of both Pol λ and Pol Δλ, but this effect was observed at micromolar concentrations of XRCC1, which were ~50 times above the K_d_ value determined for the Pol λ–XRCC1 complex, indicating the presence of another stimulatory factor aside from protein–protein interactions. By dynamic light scattering (DLS) analysis of the hydrodynamic size of proteins and by fluorescence microscopy, we found that XRCC1 undergoes microphase separation (forming its own protein-rich microdroplets) and coassembly with Pol λ in the presence of damaged DNA. Therefore, the dependence of Pol λ activity on DNA-induced XRCC1 microphase separation points to a possibility of the formation of XRCC1-related condensates associated with BER regulation.

## 2. Results

### 2.1. XRCC1 Stimulates Gap-Filling DNA Synthesis Catalyzed with Pol λ 

**Figure 1:**
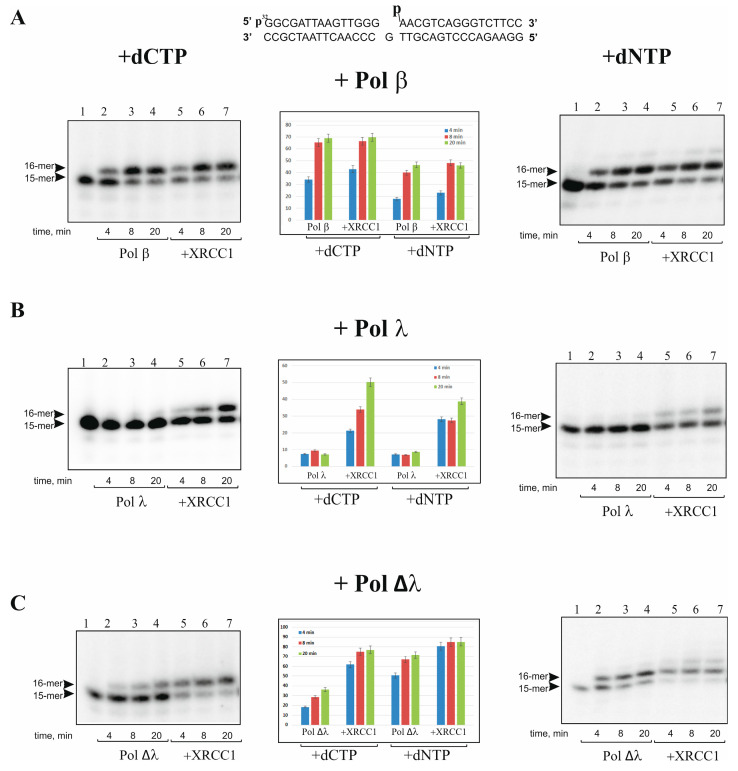
XRCC1 stimulates DNA synthesis catalyzed by Pols β, λ, or Δλ on gap-DNA mimicking short-patch BER intermediate. The DNA synthesis catalyzed by Pols β (**A**), λ (**B**), and Δλ (**C**) was carried out in reaction mixtures composed of [^32^P]labeled gap-DNA (10 nM), dCTP (50 nM), and Pol β, λ, or Δλ (5 nM). XRCC1 (500 nM) was added to the reaction mixture simultaneously with a DNA polymerase, as indicated. Lane 1 in each panel shows a control reaction without polymerases. Histograms (central panel) present the relative level of primer extension (%) (the mean ± SD of three independent experiments).

**Figure 2:**
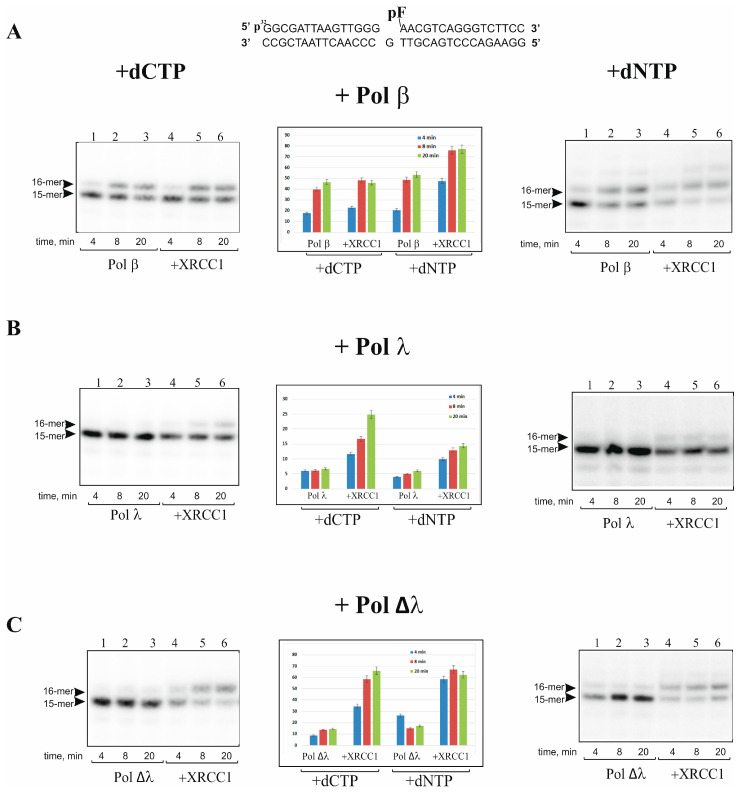
XRCC1 stimulates DNA synthesis catalyzed by Pols β, λ, and Δλ on gap-pF-DNA mimicking long-patch BER intermediate. The DNA synthesis catalyzed by Pols β (**A**), λ (**B**), and Δλ (**C**) was carried out in reaction mixtures composed of [^32^P]labeled gap-pF-DNA (10 nM), dCTP (50 nM) or dNTP (50 nM), and Pol β, λ, or Δλ (5 nM). XRCC1 (500 nM) was added to the reaction mixture simultaneously with a DNA polymerase, as indicated. Histograms (central panel) present the relative level of primer extension (%) (the mean ± SD of three independent experiments).

First, we evaluated the DNA synthesis activities of Pol λ, its mutant Pol Δλ lacking the BRCT and SPR domains (amino acid residues 1-244), and Pol β and estimated the effect of XRCC1 on the enzymatic activities. One-nucleotide-gapped DNA duplexes containing either a phosphate (p) or furan-phosphate (pF) group at the 5′-end of the gap were used as the DNA substrates gap-DNA and gap-pF-DNA, respectively. It should be noted that the 5′-pF group cannot be removed by the lyase activity of Pol β or Pol λ [40,41,42,43]; therefore, gap-pF-DNA mimics intermediates of the long-patch BER pathway, while gap-DNA is an intermediate of short-patch BER. As shown in Figure 1, the gap-filling and strand-displacement DNA synthesis by these polymerases at a low concentration (5 nM) proceeded with different efficiency. Pol Δλ, just like Pol β, was able to perform gap-filling DNA synthesis in the presence of dCTP in the case of either gap-DNA (Figure 1) or gap-pF-DNA (Figure 2), albeit with a low efficiency. By contrast, Pol λ at the concentration of 5 nM was not efficient in the gap-filling synthesis for either DNA substrate (Figure 1B and Figure 2B). We also tested the activity of Pol λ, Pol Δλ, and Pol β at a higher concentration (10 nM or 50 nM), but to analyze the influence of XRCC1 on the kinetic of DNA synthesis at the initial stage, it was necessary again to decrease the concentration of these polymerases up to 5 nM. Under the selected conditions, it turned out that Pol β and Pol ∆λ performed efficient DNA synthesis. Although Pol λ at 5 nM was practically unable to fill the gap, the XRCC1-dependent stimulation of DNA synthesis was clearly visible. Since XRCC1 is a cofactor of Pol β, it may also regulate both Pol λ and ∆λ DNA synthesis. We found that the high concentration (500 nM) of XRCC1 resulted in different effects: XRCC1 did not show a visible influence on the gap-filling activity of Pol β with either gap-DNA (Figure 1A) or gap-pF-DNA (Figure 2A) but noticeably enhanced the activity of Pol λ (~5-fold; Figure 1B and Figure S1B) and Pol Δλ (~2-fold; Figure 1C and Figure 2C). 

It was previously shown that Pol λ alone is not efficient in performing strand-displacement DNA synthesis [17,38]. Since strand-displacement DNA synthesis Pol β is stimulated by XRCC1 [26], we then examined the effect of XRCC1 on Pol ∆λ and Pol λ activities using gap-DNA and gap-pF-DNA mimicking the long-patch BER intermediates (Figure 1 and Figure 2). Under the conditions of a high concentration of XRCC1 (500 nM), we analyzed DNA synthesis catalyzed by the polymerases in the presence of four dNTPs on gap-DNA and gap-pF-DNA substrates (Figure 1 and Figure 2, right panel). At a low concentration (5 nM) of these polymerases, as expected, the one-nucleotide gap filling was mainly observed because only a minor amount of products (plus two nucleotides) was detected for Pol β and Pol Δλ (Figure 1A, Figure 2A, Figure 1C and Figure 2C, right panel). At the same time, Pol λ was not able to perform strand-displacement synthesis on the gap-pF containing DNA in the absence or presence of XRCC1 (Figure 1B and Figure 2B, right panel). 

The effect of XRCC1 on the activity of the polymerases was very similar between gap-DNA and gap-pF-DNA. In addition, in the presence of four dNTPs, Pol β was not efficient in strand-displacement DNA synthesis, and XRCC1’s stimulatory effect was more pronounced on gap-pF-DNA in comparison with gap-DNA (compare Figure 1A and Figure 2A). The results indicate that Pol λ exhibits weaker DNA synthesis compared to that of Pol β and Pol ∆λ on the one nucleotide gap containing substrate, confirming that SPR and BRCT domains functionally suppress Pol λ activity [19]. XRCC1 can strongly stimulate the activity of full-length Pol λ in gap-filling DNA synthesis, implying their interaction and/or formation of a binary (XRCC1–Pol λ) or ternary (Pol λ–XRCC1–DNA) complex similar to a known interaction of XRCC1 with Pol β [31].

### 2.2. Pol λ Binds the C-Terminal Region of XRCC1

According to previously reported data, XRCC1 forms a stable complex with Pol β and modulates the catalytic activity of the polymerase [31,44]. Thus, the observed stimulation of DNA synthesis activity of Pol λ by XRCC1 may indicate the formation of a complex between the proteins. To test this hypothesis, we employed fluorescence resonance energy transfer (FRET) experiments to detect and quantitatively characterize XRCC1 interaction with full-length Pol λ or its truncated mutant (Δλ) in comparison with Pol β. For this purpose, we utilized Cy3-labeled XRCC1 and a Cy5-labeled polymerase (Pol β, Pol λ, or Pol Δλ) to detect the protein–protein complex formation. Fluorophores Cy3 and Cy5 are typically used as a donor–acceptor pair in FRET experiments that allow the detection of direct physical interactions between proteins [45].

**Figure 3:**
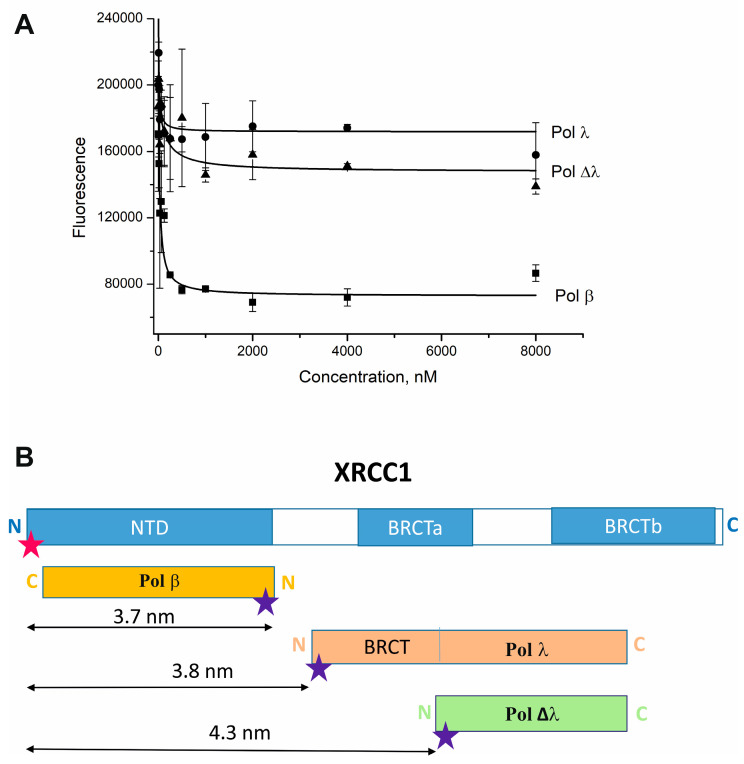
(**A**) Quantitative analysis of XRCC1–Polymerase interaction by FRET. The Cy3-labelled XRCC1 (30 nM) was excited at 530 nm in the absence or presence of increasing concentration (30–8000 nM) of the Cy5-labelled Pol β (black squares), Pol ∆λ (black triangles) or Pol λ (black circles). The relative fluorescence intensities were monitored at 580 nm. Dichroic filter 552.5 nm. Typical titration curves are shown. Fluorescence intensity, units along the Y axis. (**B**) A proposed model of the XRCC1 interaction with DNA polymerases. A schematic representation of the domain structure of XRCC1 and DNA polymerases, where N and C denote the N- and C-terminus of proteins, respectively. Asterisks indicate fluorescent labels Cy3 (red) and Cy5 (blue). The distance (R) between the donor and acceptor presented in Table 1 is depicted by arrows.

In the FRET assays, values of the binding parameters (equilibrium constant, K_d_) and FRET efficiency (E) determined from these experiments indicated a direct interaction between XRCC1 and each DNA polymerase (Table 1, Figure 3A). 

A comparison of XRCC1’s affinity for these polymerases revealed clear differences in K_d_ values; namely, XRCC1 has a nearly 3.0-fold stronger affinity for Pol λ (~9.6 nM) than for Pol β (~29 nM). Nonetheless, FRET efficiency (E values in Table 1) was very similar among the polymerases. Based on these data, a difference in the mode of XRCC1 interaction with Pol λ and Pol β can be supposed. The main difference between the two DNA polymerases is the presence in Pol λ of a non-catalytic N-terminal region, which includes the SPR domain, which is supposed to be a target for post-translational modification and BRCT domain taking part in protein–protein interactions [7,13,46]. Indeed, deletion of the BRCT and SPR domains in Pol λ significantly weakened the binding affinity between the polymerase and XRCC1; for instance, K_d_ of the Pol λ–XRCC1 complex is 15-fold lower than that of the Pol Δλ–XRCC1 complex (~9 and ~139 nM, respectively) (Table 1). The N-terminal domain of XRCC1 was shown to interact with the C-terminal palm-thumb domain of Pol β [47]. If the interaction with XRCC1 with Pol λ is mediated via a similar domain (aa 387-575), the N-terminal deletion (aa 1-244) of the polymerase will not strongly influence XRCC1-polymerase interactions. The significant differences in K_d_ values (Table 1) indicated that the N-terminal non-catalytic domain of Pol λ is most probably involved in the interaction with XRCC1. 

The distance (R) between the Cy3 residue at the N-terminus of XRCC1 and Cy5 at the N-terminus of the DNA polymerases was also compared (Table 1, Figure 3B). 

The R values for Cy5-Pol λ-Cy3-XRCC1 and Cy5-Pol ∆λ–Cy3-XRCC1 pairs were different and reflect changes in the molecular distance between the interacting donor and acceptor fluorophores in the case of deletion of N-terminus of Pol λ. Although there were no structural data for full-length XRCC1 [48], the prolate ellipsoid model based on biophysical studies for the three-dimensional shape of XRCC1 in solution was supposed [49]. Thus, XRCC1 can be shown as an extended rod-like structure consisting of two BRCT domains (BRCTa and BRCTb), two interdomain linker regions, and the N-terminal domain (NTD) that participates in the Pol β interaction (Figure 3B) [30,48,49]. The C-terminal domain of Pol β is reported to interact with the N-terminal NTD of XRCC1 [50,51], and according to our R values obtained by FRET (Table 1), these proteins may be arranged in an “antiparallel” orientation, so that the N-terminal domain of XRCC1 is close to the C-terminal domain of the polymerase (Figure 3B). Taking into account that the R values of polymerases β and λ were similar (Table 1), Pol λ most likely interacts with XRCC1 in a “parallel” orientation so that the N-terminal BRCT domain of Pol λ interacts with the central region of XRCC1 that includes the BRCTa domain. In support of this hypothesis, the deletion of the N-terminal domains in Pol λ resulted in an increase in R value (Table 1), suggesting that Pol Δλ is located by 0.6 nm farther away from the N-terminus of XRCC1 (Figure 3B). Thus, we suggested that the major region responsible for the interaction with Pol λ is within the central region of XRCC1, including BRCT domains.

### 2.3. XRCC1 Can Form Microphases with DNA and DNA Polymerases

**Figure 4:**
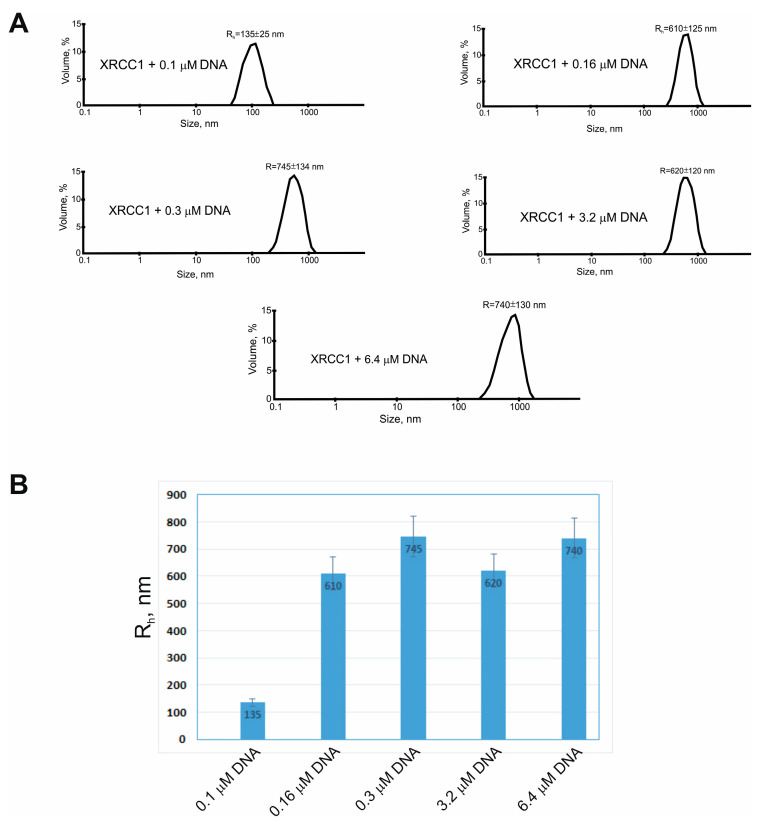
XRCC1 forms protein-rich microphases in the presence of gap-DNA. Depicted are typical volume-weighted size distributions for 2.5 µM XRCC1 with different concentrations of gap-DNA (**A**). The profiles were obtained by means of experimental autocorrelation functions in the Zetasizer Nano ZS software. The hydrodynamic radius of XRCC1 (R_h_, nm) was determined in reaction mixtures consisting of 2.5 µM XRCC1 and 0.1–6.4 µM gap-DNA. Histograms (**B**) present the hydrodynamic radius (R_h_, nm) for XRCC1. R_h_ is the average value from at least three DLS experiments.

**Figure 5:**
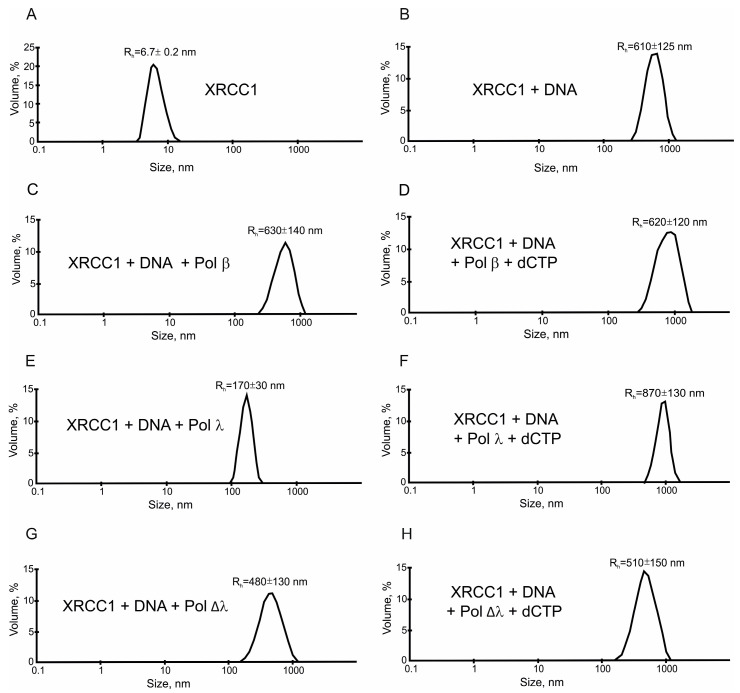
Pol λ influences the formation of protein-rich microphases by decreasing the size of XRCC1–DNA higher-order assemblies. Depicted are typical volume-weighted size distributions for 2.5 µM XRCC1 alone (**A**), for XRCC1 in the presence of 0.16 µM gap-DNA (**B**), after t 0.25 µM Pol β addition (**C**) and after 0.1 mM dCTP and 5 mM Mg^2+^ addition (**D**), after 0.25 µM Pol λ addition (**E**) and after 0.1 mM dCTP and 5 mM Mg^2+^ addition (**F**), after 0.25 µM Pol Δλ addition (**G**), and after 0.1 mM dCTP and 5 mM Mg^2+^ addition (**H**). The profiles were obtained by means of experimental autocorrelation functions in the Zetasizer Nano ZS software. The average hydrodynamic radius (R_h_) computed from the distributions is presented as well. R_h_ is the average value from at least three DLS experiments.

Although FRET-based quantification of Pol λ–XRCC1 interactions showed that XRCC1 interacts with Pol λ with strong affinity (K_d_~9 nM) (Table 1), it was unable to stimulate Pol λ activity at the nanomolar concentration of the proteins. Both gap-filling and strand-displacement activities of Pol λ were strongly enhanced by XRCC1 when its concentration was raised up to 500 nM (Figure 1B). This outcome indicated that in addition to the XRCC1–Pol λ protein–protein interactions, another factor may exist that contributes to the stimulatory effect. One of the possible explanations is that XRCC1 may form protein-rich microphases in the presence of DNA duplexes (gap-DNA or gap-pF-DNA) used for the testing of the polymerase activity because we have previously demonstrated that XRCC1 undergoes condensation, i.e., forms protein-rich microphases in the presence of a nucleic-acid-like polymer of ADP-ribose: PAR [52]. Therefore, we attempted to evaluate the possibility of XRCC1 condensation in a reconstituted system that included gap-DNA and a DNA polymerase. By DLS and fluorescence microscopy, we characterized the phase behavior of XRCC1 in the presence of gap-DNA and/or one of the following DNA polymerases: Pol β, Pol λ, or Pol Δλ. First, the hydrodynamic radius (R_h_) of XRCC1 was measured in the absence or presence of gap-DNA and after the addition of a polymerase (Pol β, Pol λ, or Pol Δλ) (Figure 4 and Figure 5).

In each case, volume-weighted distributions (%) of particle size had one peak (Figure 4A), suggesting that the protein solution was homogeneous. In the absence of DNA, XRCC1 itself formed only particles with a R_h_ value of approximately 6.7 nm (which can be most likely attributed to the protein homodimer [27]) and was unable to form higher-order structures (Figure 5A). Upon the addition of DNA at an elevated concentration, XRCC1 higher-order assemblies with a size of ~135 to ~740 nm were detected (Figure 4). Consequently, gap-DNA is able to induce the formation of protein-rich phases or higher-order assemblies in a solution of the XRCC1 protein, and this process is affected by the concentration of DNA. It seems that XRCC1–DNA assembly is based on the DNA-binding ability and BRCT-mediated self-assembly of the protein [34,53]. In this context, DNA polymerases having a DNA-binding activity and interacting with XRCC1 can affect the XRCC1–DNA microphase separation. To determine whether DNA polymerases influence the higher-order assembly of XRCC1, we carried out a DLS for XRCC1 analysis size in the presence of gap-DNA and a polymerase (Figure 5). The addition of Pol β did not influence the XRCC1–DNA higher-order assembly (Figure 5C), and large particles with an average R_h_ ~ 630 nm were observed. Upon the addition of dCTP to initiate DNA synthesis, the size of the particles was practically unchanged (Figure 5D).

When Pol λ was added to the XRCC1–DNA mixture, the average size of particles (R_h_) decreased from ~610 to ~170 nm (Figure 5E). It is possible that more effective XRCC1–Pol λ complex formation (Table 1)—in comparison with Pol β and Pol Δλ—leads to a rearrangement of the intermolecular interactions of XRCC1 and a change in the size of the particles. Indeed, the DNA binding ability of Pol λ was shown to rely on a Pol β-like domain [17]; thus, the C-terminal truncated form and the full length of Pol λ are expected to have a similar DNA binding affinity. Then, the full-length Pol λ possessing BRCT and SPR domains, through which it could potentially interact with the XRCC1 (Table 1), can perturb the self-assembly of XRCC1 and form binary and/or ternary (XRCC1-Pol λ-DNA) complexes, thereby reducing particles size. When dCTP was added to the XRCC1-Pol λ-DNA mixture, large particles (R_h_ ≈ 870 nm) were detectable again (Figure 5F). Most likely, the gap in the DNA duplex was filled by Pol λ, after which Pol λ left the nicked DNA and ternary complexes were disrupted, thereby leading to the rearrangement of XRCC1 intermolecular interactions again, and the size of XRCC1–DNA assemblies enlarged. 

When Pol Δλ was added to the XRCC1–DNA mixture, a small decrease in the size of the associates was observed (Figure 5G), possibly due to the low-affinity interaction between XRCC1 and Pol Δλ (Table 1). No changes were observed after the addition of dCTP (Figure 5H).

Thus, Pol λ appears to influence XRCC1–DNA microphase separation by forming binary Pol λ-XRCC or ternary Pol λ-XRCC-gapped DNA complexes, thereby affecting the size of the condensates. Note also that none of the tested polymerases were able to fully disrupt the XRCC1 assemblies (Figure 5).

**Figure 6:**
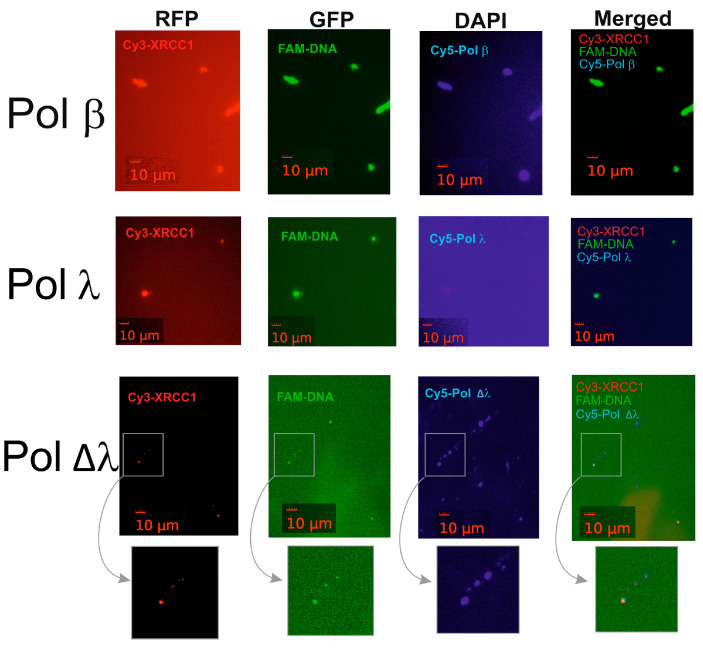
Fluorescent detection of protein-rich microphases arising after the XRCC1 interaction with gap-DNA in the presence of a DNA polymerase. The biomolecules labeled with different fluorophores (Cy3-XRCC1, Cy5-DNA-Pol, and FAM-DNA) were visualized in images acquired with appropriate filters (RFP for Cy3, GFP for FAM, and Cy5 for Cy5); the overlapping signals of distinct fluorophores were visualized in a superimposed image (merged). Scale bar: 10 µM. Preparation of the samples is detailed in Materials and Methods.

To visualize the coassembly of XRCC1 with gap-DNA and a DNA polymerase (β, λ, or Δλ) as detected by DLS, we attempted to apply fluorescence microscopy to the proteins and DNA labeled with different fluorophores. For this purpose, we utilized Cy3-labeled XRCC1, FAM-labeled DNA, and a Cy5-labeled polymerase (β, λ, or Δλ) and assessed the formation of XRCC1–DNA condensates under conditions of addition of different polymerases (Figure 6).

Images revealing the presence of fluorescent signals were obtained for the mixture of Cy3-XRCC1, FAM-gap-DNA, and Cy5-Pol β, λ, or ∆λ (Figure 6). The images were captured and superimposed on each other to obtain a multifluorescent merged image (see Figure 6, the fourth picture in each row). Detected spots in the combined images showed an overlap of fluorescent signals specific to all three labeled components, indicating the formation of higher-order assemblies and colocalization of XRCC1 with gap-DNA and a DNA polymerase.

In summary, these data suggest that Pol β and λ may be concentrated within XRCC1–DNA condensates.

## 3. Discussion

The multistep BER process requires coordinated actions of multiple enzymes that catalyze the individual steps. Proposed coordinated models of BER involve either the “passing the baton” mechanism, including the transfer of a DNA repair intermediate from one enzyme to another, or the formation of transient damage-specific complexes containing enzymes required for the repair of a given DNA lesion [54,55,56]. Protein–protein interactions also contribute to sequential repair reactions in this process [31,57]. Both models include the assembly of multiprotein complexes coordinated by scaffold proteins such as XRCC1 or PARP1 (PARP2) [57,58,59]. BER and SSB repair are tightly regulated by XRCC1 [60,61,62]. Nevertheless, the critical role of XRCC1 in BER is not fully elucidated.

In this work, we examined the DNA synthesis activity of Pol λ and its truncated form Pol Δλ with XRCC1 scaffold protein involved in BER regulation in comparison with Pol β. DNA polymerase λ belongs to the same structural X family as Pol β, possesses the same enzymatic activities, i.e., DNA polymerase and 5′-dRP lyase, and effectively performs gap-filing DNA synthesis. Therefore, Pol λ can function as a backup polymerase in BER [10,11,12]. The main difference between the two Pols is the presence in the Pol λ structure of additional proline-serine-rich (SPR) and N-terminal BRCT domains [8,9]. Although Pol λ-dependent DNA repair synthesis has been intensively studied [7,10,11,12,36,37,38,39], the functional interaction between Pol λ and other BER factors is still far from elucidation. We found that full-length Pol λ interacts with XRCC1 with strong affinity, but the affinity of XRCC1 for Pol Δλ proved to be weaker, suggesting that the Pol λ BRCT and SPR domains play a pivotal part in the interaction with the BER scaffold protein XRCC1. Moreover, XRCC1 stimulates gap-filling activity of Pol λ stronger than Pol Δλ (Figure 1 and Figure 2). 

The understanding of the regulation of DNA repair systems has seen a revolution in the last few years owing to the discovery of biomolecular condensates generated with the participation of multivalent biomolecules via a process of liquid–liquid phase separation (LLPS) [63,64,65,66,67]. DNA repair foci seem to be associated with the accumulation of repair proteins at the damage site and recently were recognized as a protein condensate formed at a DNA damage site [64,65,66]. A special role in the formation of BER-related condensates belongs to PAR and intrinsically disordered FET family proteins (FUS, EWS, and TAF15), which can interact with PARylated PARP1 activated at a site of a DNA lesion and form DNA repair compartments [64,65,67]. Another protein that performs regulatory and coordinative functions in BER/SSB repair is XRCC1 [32]. XRCC1 was previously shown to form protein-rich microphases in the presence of PAR in vitro [52]. 

**Figure 7:**
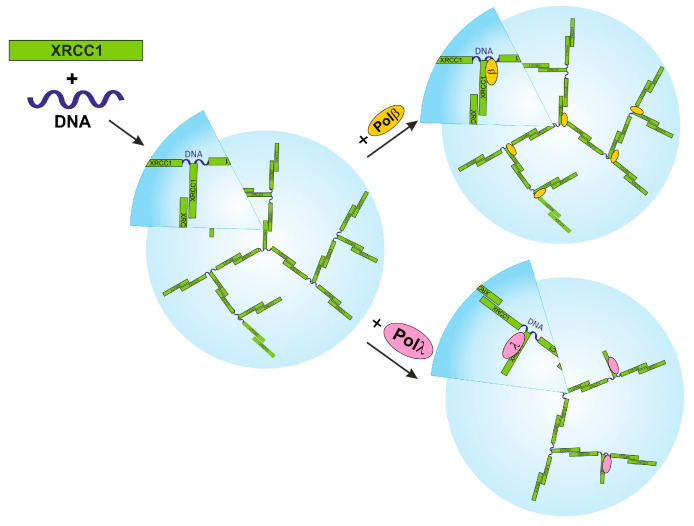
The proposed model of XRCC1 microphase separation in the presence of DNA and a DNA polymerase β or λ.

In the current study, we show that XRCC1 at micromolar concentrations undergoes microphase separation in the presence of DNA duplexes. Phase separation of proteins is believed to be promoted by multivalent interactions that can be achieved through protein self-association and/or binding to other biomolecules such as a nucleic acid or protein partner [68]. From this point of view, XRCC1 has an ability to form homodimers mainly via the BRCTb domain and heterodimers through the N-terminal domain and BRCT domains, interacting with a large number of BER proteins [27,30,33,34,44,69]. Several research articles have also highlighted an ability of XRCC1 to interact in vitro with nicked and gapped DNA via the N-terminal domain and BRCTa [49,53,70]. Thus, these two biochemical properties of XRCC1, namely, DNA binding and homo- and hetero-association, enable the formation of protein–DNA-rich phases. Indeed, XRCC1 at micromolar concentrations is reported to exist predominantly as a dimer in solution [27]; thus, the oligomeric form of XRCC1 may also bind to DNA molecules and/or form bridges between two distinct DNA molecules. Such multivalent interactions involving the simultaneous emergence of multiple supramolecular bonds, such as XRCC1–XRCC1 and XRCC1–DNA, may underlie the DNA-dependent condensation of the protein (Figure 7). The addition of Pol β did not disrupt the supramolecular bonds XRCC1–XRCC1 and XRCC1–DNA, whereas the addition of Pol λ led to partial severance of these bonds and, consequently, to a decrease in the size of the condensates (Figure 7).

The condensation of XRCC1 can further promote the concentration of other partner proteins on the basis of their ability to interact with the scaffold. Accordingly, we found that XRCC1 condensation is also triggered by DNA duplexes in the presence of a DNA polymerase. In support of this notion, we demonstrated that XRCC1–DNA polymerase co-condensation is accompanied by enhancement of gap-filling and strand-displacement activities of Pol β and λ (Figure 1 and Figure 2). Earlier, it was revealed that XRCC1 binds to Pol β with strong affinity and stimulates Pol β activity owing to XRCC1–Pol β–DNA complex formation [26,31]. Our data also point to the strong-affinity interaction of XRCC1 with Pol λ and much weaker affinity for the truncated Pol λ’s lack of the BRCT and SPR domains (Table 1). Since the SPR domain is mainly considered as a target for post-translational modification [25], it is possible that the Pol λ BRCT domain mediates Pol λ’s interactions with XRCC1 (Figure 3B) and suggests that the XRCC1–Pol λ complex may exert a regulatory effect on polymerase functions. Nevertheless, under conditions favoring the formation of the XRCC1–Pol λ complex, i.e., when XRCC1 concentration is similar to or moderately higher than the K_d_ value, we observed no influence of XRCC1 on the activity of full-length Pol λ.

Thus, we propose that there is no clear correlation between XRCC1’s binding affinity for DNA polymerases and XRCC1’s capacity to stimulate the activity of these polymerases under our experimental conditions. Indeed, XRCC1 manifested strong affinity for full-length Pol λ and bound to the truncated form (Pol Δλ) with weak affinity (K_d_ of 9.6 and 139 nM, respectively), whereas in the absence of XRCC1, gap filling proceeded slowly for both polymerases, but the DNA synthesis was promoted by XRCC1 with similar efficiency (~5-fold for Pol λ and 2-fold for Pol Δλ) (Figure 1 and Figure 2).

Consistently with the idea that the higher-order assembly of XRCC1–DNA is responsible for the stimulation of DNA synthesis activity of these polymerases, the polymerases did not disrupt the XRCC1–DNA assemblies in the absence or presence of dCTP, which is required for DNA synthesis (Figure 5). Therefore, XRCC1–DNA assemblies act as scaffolds, pulling polymerases interacting with XRCC1 and/or gapped DNA into the microphase.

This notion raises the question of whether XRCC1 condensation occurs at DNA repair sites and what functional advantage could be conferred by such condensation. Although most of the cell experiments have proven a poly(ADP-ribose)-dependent formation of XRCC1-containing foci [71,72,73,74], which is in agreement with the formation of XRCC1- poly(ADP-ribose)-microphases in vitro, there are data that XRCC1 alone can form repair foci, for example, after endogenous DNA damage or at a low level of induced DNA damage [75] or after poly(ADP-ribose)-degradation by PARG, when XRCC1 could bind directly to the DNA damage site and act as a scaffold promoting the formation of a transient damage-specific repair complex [76]. 

Thus, BER proteins that bind to XRCC1 could be selectively enriched within XRCC1 condensates, while noninteracting proteins will be excluded. Furthermore, DNA lesion–binding proteins are also able to enter condensates formed by XRCC1. Overall, XRCC1 can be considered a repair scaffold molecule that can potentially generate repair condensates and regulate a specific protein’s recruitment during BER and/or SSB repair. High-affinity interaction of Pol λ with XRCC1 and the recruitment of Pol λ to the XRCC1-rich microphases extend the ability of Pol λ to deal with BER-related tasks when the Pol λ–XRCC1 interaction may underlie alternate DNA repair synthesis in the event that Pol β is unable to conduct DNA synthesis.

Additional research will clarify whether XRCC1 condensation and XRCC1’s interaction with Pol λ identified here can occur under physiological conditions and have functional consequences such as BER and/or SSB repair regulation.

## 4. Materials and Methods

### 4.1. Reagents, Plasmids, and Proteins 

Radioactive [γ-^32^P]ATP (5000 Ci/mmol) was produced in the Laboratory of Radiochemistry (ICBFM SB RAS, Novosibirsk, Russia); phage T4 polynucleotide kinase was purchased from Biosan (Novosibirsk, Russia) and stained molecular mass markers were from Fermentas (Vilmius, Lithuania), whereas reagents were for electrophoresis and buffer components from Sigma (Saint Louis, MO, USA). Ultrapure dNTPs and dCTP were acquired from SibEnzyme (Novosibirsk, Russia), and sulfo-cyanine 5 NHS ester and sulfo-cyanine 3 NHS ester from Lumiprobe (Moscow, Russia). Oligodeoxynucleotides were synthesized in the Laboratory of Biomedical Chemistry (ICBFM SB RAS).

The recombinant plasmid DNA coding for XRCC1 was a kind gift from Dr. Keith W. Caldecott (University of Sussex, Brighton, UK). The recombinant plasmid DNAs encoding human Pol λ or rat Pol β were a kind gift from Dr. S.H. Wilson (NIEHS, USA). 

His-tagged full-length Pol λ was expressed in *Escherichia coli* (*E.coli*) BL21(DE3) RIL cells and purified as described before [16], with some modifications. Briefly, Pol λ was purified by Ni-NTA agarose affinity chromatography, P11 phosphocellulose chromatography, and Heparin Sepharose chromatography. Rat recombinant Pol β was expressed in *E. coli* BL21(DE3) and purified as described elsewhere [77]. The construction of the recombinant plasmid DNA coding for the truncated form of human Pol λ (Pol Δλ) has been described previously [36]. His-tagged Pol Δλ was expressed in *E. coli* BL21(DE3) RIL and purified to homogeneity using the same purification stages as those for full-length Pol λ, except that P11 phosphocellulose chromatography was not employed. His-tagged XRCC1 was overexpressed in the Rosetta 2(DE3)pLysS strain of *E. coli* and purified as previously described [78] with some modifications. Briefly, XRCC1 was purified by Co^2+^-IMAC affinity chromatography, heparin affinity chromatography, and mono Q anion exchange chromatography. 

### 4.2. Radioactive Labeling of Oligonucleotides and Preparation of DNA Duplexes

Oligodeoxynucleotides were 5′-[^32^P]labeled with T4 polynucleotide kinase and [γ-^32^P]ATP as described in ref. [79]. Unreacted [γ-^32^P]ATP was removed by passing the mixture through a MicroSpin^TM^ G-25 column (Amersham, Piscataway, NJ, USA). Complementary oligodeoxynucleotides were annealed in equimolar amounts by heating a solution at 95 °C for 5 min, followed by slow cooling to room temperature.

The sequences of the oligonucleotides and names of DNA duplexes used in the experiments were as follows: 

gap-DNA 5′-^32^pGGCGATTAAGTTGGG **p**AACGTCAGGGTCTTCC-3′

3′-CCGCTAATTCAACCC GTTGCAGTCCCAGAAGG-5′

gap-pF-DNA 5′-^32^pGGCGATTAAGTTGGG **pF**AACGTCAGGGTCTTCC-3′

3′-CCGCTAATTCAACCC G TTGCAGTCCCAGAAGG-5′

Where p—5’-phosphate, pF—5’-furan phosphate.

### 4.3. The DNA Synthesis Assay

The reaction mixtures (10 μL) composed of 10 nM 5′-[^32^P]labeled gap-DNA or gap-pF-DNA, 50 mM Tris-HCl (pH 7.5), 5 mM Mg^2+^, 0.5 mM DTT, 0.25 mg/mL BSA, 50 nM dCTP (or dNTP), and 5 nM one of DNA polymerases (β, λ, or Δλ) were incubated at 37 °C for different time (4, 8, or 20 min). XRCC1 (500 nM) was added to the reaction mixture simultaneously with a DNA polymerase, as indicated in the figure legends. Then, the reactions were terminated by the addition of a formamide dye and heating for 5 min at 95 °C. The products were analyzed by electrophoresis in a 20% polyacrylamide gel with 8 M urea, followed by phosphorimaging using a Biomolecular Imager Typhoon FLA 9500 (GE Healthcare, Chicago, IL, USA).

### 4.4. Fluorescent Labeling of XRCC1 and Pol β, λ, and Δλ 

NHS-esters of sulfo-Cyanine 3 (Cy3-SE; Lumiprobe) or sulfo-Cyanine 5 (Cy5-SE; Lumiprobe) were dissolved in dimethyl sulfoxide up to 10 mM concentration and used for protein labeling. For N-terminal protein labeling, the reaction mixture consisted of 50 mM HEPES-NaOH (pH 8.0), 100 mM NaCl, 2 mM EDTA, 10% glycerol, 10 µM protein, and a 10-fold molar excess of the reagent over the protein. 

The reaction was carried out overnight at 4 °C in the dark and stopped by the addition of ethanolamine to a final concentration of 2 mM. The unreacted Cy3 or Cy5 dyes were removed via dialysis against a buffer composed of 50 mM HEPES-NaOH (pH 8.0), 100 mM NaCl, 2 mM EDTA, 10% glycerol, and 2 mM DTT, followed by concentrating the Cy3-labeled XRCC1 or Cy5-labeled DNA polymerases in ultrafiltration spin columns. The degree of protein labeling was calculated via this equation:

Degree of labeling = A_max_ × ε_prot_/([A_280_ − A_max_] × CF_280_ × ε_280_)

A_max_ and A_280_ were determined spectrophotometrically on a CLARIOstar microplate spectrometer (BMG Labtech GmbH, Ortenberg, Germany), the extinction coefficient of each protein (ε_prot_) was based on Expasy Protparam Data, and the extinction coefficients (ε_280_) for Cy5 and Cy3 were taken from Lumiprobe protocols. Correction factor (CF_280_) 0.06 for Cy3 and 0.04 for Cy5. The degree of labeling was estimated at 40% for XRCC1, 25% for Pol β, 15% for Pol λ, and 26% for Pol ∆λ.

### 4.5. FRET Measurements 

Reaction mixtures consisted of 50 mM HEPES-NaOH (pH 8.0), 100 mM NaCl, 2 mM DTT, 30 nM Cy3-labeled XRCC1, and 0–8000 nM Cy5-labeled Pol β, λ, or Δλ. The mixtures were incubated at 25 °C for 10 min. 

Fluorescence measurements were performed at 25 °C on the CLARIOstar microplate spectrometer (BMG Labtech GmbH) in a 384-well low-volume black round-bottom polystyrene NBS microplate (Corning, Corning, NY, USA). The fluorescence intensity of the Cy3 fluorophore was detected at an excitation wavelength of 530–20 nm and emission wavelength of 580–30 nm, with a Dichroic filter of 552.5 nm. 

FRET means the quantum effect between a given pair of fluorophores, i.e., a fluorescent donor and acceptor, in which when the donor is excited, energy is transferred from the donor to the acceptor through dipole–dipole interaction [80]. FRET is characterized by energy transfer efficiency (E), which is a function of the inverse sixth power of the distance (R) between two fluorophores. The distance at which energy transfer is 50% is known as the Förster distance (R_0_). R_0_ depends on the degree of spectral overlap between the donor’s emission and the acceptor’s absorption and is a unique property of each FRET pair. 

FRET efficiency (E) was calculated by means of a fractional decrease in donor fluorescence intensity owing to the presence of the acceptor:

E = 1 − F_da_/F_d_,

where F_da_ and F_d_ are the donor’s fluorescence intensities with and without the acceptor, respectively.

K_d_ was calculated using the equation

F = F_d_ + (F_da_ − F_d_)/[1 + (K_d_/C)],

where F is the measured fluorescence intensity of a labeled protein, and F_da_ and F_d_ are the donor’s fluorescence intensities with and without the acceptor, respectively. C is protein concentration.

According to Förster theory about nonradiant energy transfer, the distance (R) between the donor and acceptor can be calculated as

$$R=R_{0}{\left(\frac{1}{E}-1\right)}^{1/6}$$

where R_0_ is the Förster distance at which the energy transfer efficiency is 50%. At R_0_ = 5.4 nm for pair Cy3–Cy5 [45], we calculated the distance (R) between Cy3 at the N-terminus of labeled XRCC1 and Cy5 at the N-terminus of labeled Pol β, λ, or Δλ. All experiments were conducted at least three times.

### 4.6. The Fluorescence Microscopy Assay

Microscopic analysis of mixture of XRCC1, gap-DNA, Pol β, λ, or Δλ was carried out by means of a CELENA^®^ S Digital Imaging System. For fluorescence microscopy, Cy3-XRCC1, Cy5-Pol β, λ, or Δλ, and FAM-labeled gap-DNA were used. 

The sequences of the oligonucleotides used in the experiment were as follows: 

FAM-gap-DNA 5′-FAM-GGCGATTAAGTTGGG **p**AACGTCAGGGTCTTCC-3′

3′-CCGCTAATTCAACCC GTTGCAGTCCCAGAAGG-5′

Where p—5’phosphate

Reaction mixtures (20 µL) were composed of a buffer (50 mM HEPES-NaOH [pH 8.0], 100 mM NaCl, and 2 mM DTT), 2.5 µM Cy3-XRCC1, 0.2 µM FAM-gap-DNA, and 2.5 µM Cy5-Pol β, λ, or Δλ, as indicated in figure legends. The samples were incubated for 10 min at 25 °C. Then, PEG 20K was added to a final concentration of 1%, and the reaction mixture aliquot (6 µL) was placed between a microscope slide and 0.17 mm coverslip. The samples were visualized with Plan Apochromat Fluor 40 X objectives with GFP, RFP, or Cy5 filters for FAM-gap-DNA, Cy3-XRCC1, and Cy5-labeled Pol β, λ, or Δλ, respectively.

### 4.7. DLS Measurements

DLS processes time-dependent fluctuations of scattered light to obtain the hydrodynamic radius (R_h_) of diffusing particles in solution at true equilibrium. DLS measurements were performed at 25 °C using a ZEN 2112 low-volume quartz cuvette on a Zetasizer Nano ZS (Malvern Instruments Ltd., Malvern, UK). All stock solutions of proteins and DNA were pre-ultrafiltered through a polyethersulfone membrane (300,000 MWCO PES pore size) in a Vivaspin centrifugal concentrator (Sartorius, Göttingen, Germany). 

XRCC1 hydrodynamic size assays in the absence or presence of DNA and a DNA polymerase (β, λ, or Δλ) were performed in reaction mixtures composed of 50 mM Tris-HCl (pH 7.5), 100 mM NaCl, 2.5 µM XRCC1, 0.16 µM gap-DNA, 0.25 µM Pol β, Pol λ, or Pol Δλ, 5 mM MgCl_2_, and 1 mM dCTP as indicated in figure legends.

The samples were equilibrated for 1 min prior to the R_h_ measurement. In the case of dCTP addition, R_h_ measurement was performed immediately after the addition in kinetic mode. All experiments were conducted at least three times. Data processing was performed as described elsewhere [27].

## Figures and Tables

**Table 1:** K_d_ values of XRCC1 complexes with DNA polymerases as determined by FRET.

Labeled Protein	K_d_, nM	E	R, nm
Cy5-Pol β	29 ± 12	0.91 ± 0.06	3.7 ± 0.2
Cy5-Pol λ	9.6 ± 4.0	0.90 ± 0.05	3.8 ± 0.2
Cy5-Pol Δλ	139 ± 14	0.80 ± 0.05	4.3 ± 0.3

The titration experiments were conducted at a constant concentration of Cy3-XRCC1 (30 nM). Values are the mean ± SD of three independent experiments.

## Data Availability

All data necessary to reproduce our results are included in this published article. Raw data are available upon request.

## Supplementary Materials

The following supporting information can be downloaded at: https://www.mdpi.com/article/10.3390/ijms25136927/s1.
