# Supplementary material for: DNA Repair Protein XRCC1 Stimulates Activity of DNA Polymerase λ under Conditions of Microphase Separation
Source: Int J Mol Sci. 2024 Jun 25;25(13):6927. doi: 10.3390/ijms25136927 (PMC11241748; doi:10.3390/ijms25136927)
Supplement: Supplementary file 1 [file ijms-25-06927-s001.zip › ijms-3024898-supplementary.pdf]

Supplementary Materials

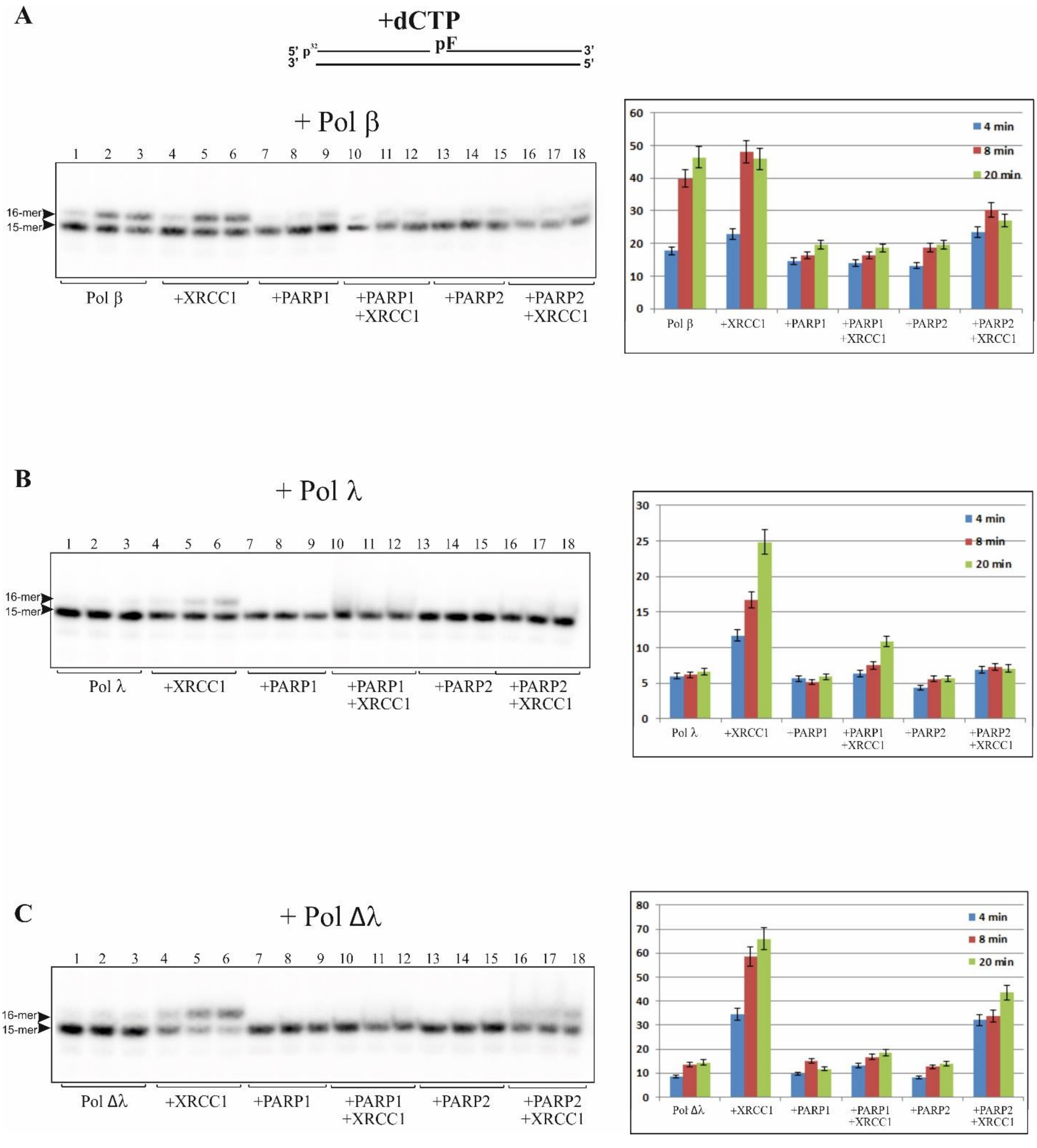

Fig. S1. The activities of Pols β, λ and Δλ in 5′-pF-gap-filling reaction in the presence of XRCC1 and PARP1 or PARP2. The reaction mixtures contained DNA duplexes (10 nM), dCTP (50 nM) and Pol β, λ or Δλ (5 nM). XRCC1 (500 nM) or PARP1 or PARP2 (50 nM) was added to the reaction mixture simultaneously with DNA-polymerases as indicated. Lane 1 in each panel shows a control reaction without polymerases. On the right side of each autoradiograph is a histogram, which graphically reflects the influence of XRCC1 and PARP1 or PARP2 on the activity of DNA polymerases. The histogram was plotted from the results of three experiments.

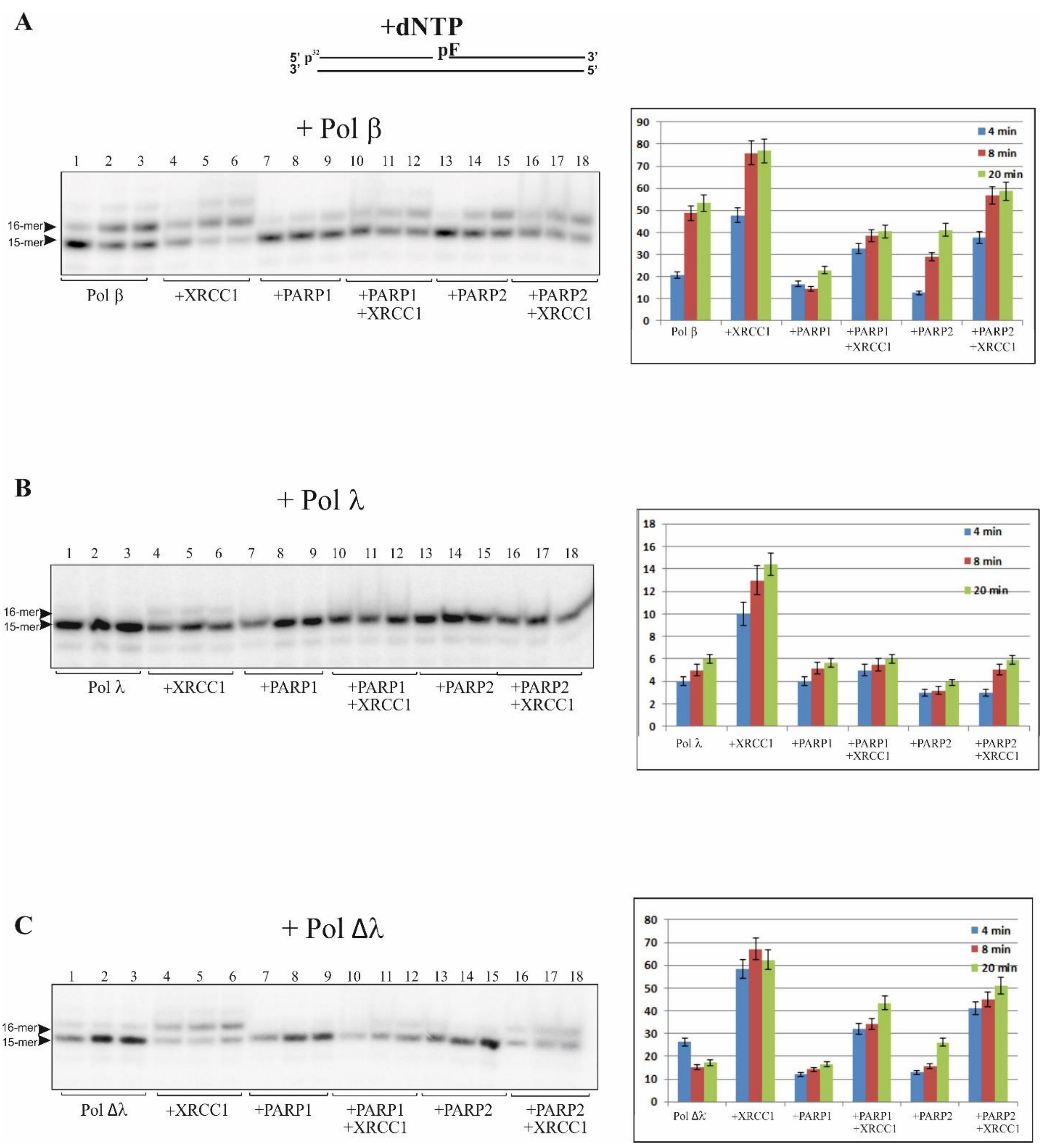

Fig. S2. Strand displacement synthesis by Pols β, λ and Δλ in 5′-pF-gap-filling reaction in the presence of XRCC1 and PARP1 or PARP2. The reaction mixtures contained DNA duplexes (10 nM), dNTP (50 nM) and Pol β, λ or Δλ (5 nM). XRCC1 (500 nM) or PARP1 or PARP2 (50 nM) was added to the reaction mixture simultaneously with DNA-polymerases as indicated. Lane 1 in each panel shows a control reaction without polymerases. On the right side of each autoradiograph is a histogram, which graphically reflects the influence of XRCC1 and PARP1 or PARP2 on the activity of DNA polymerases. The histogram was plotted from the results of three experiments.

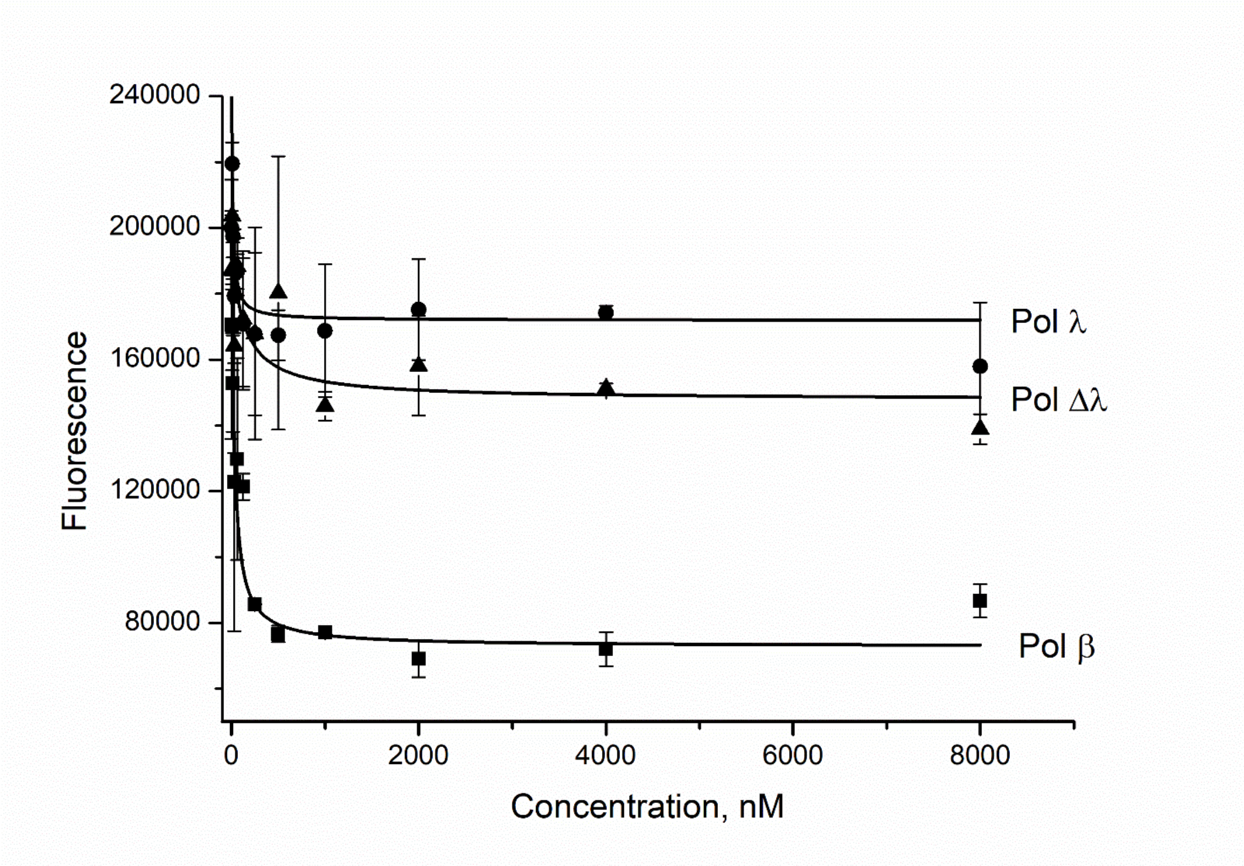

Fig. S3. Detection of XRCC1 complexes with DNA polymerases by fluorescence titration. The Cy3-labelled XRCC1 (30 nM) was excited at 530 nm in the absence or presence of increasing concentration of the Cy5-labelled DNA-polymerases. The relative fluorescence intensities were monitored at 580 nm. Dichroic filter 552.5 nm. Typical titration curves are shown.
